# Single stage direct -to- implant breast reconstruction following mastectomy (The use of Ultrapro® Mesh)

**DOI:** 10.1186/s12957-024-03576-2

**Published:** 2024-11-12

**Authors:** Maher H. Ibraheem, Omnia Mohammed Mohammed Essawy, Inas Moaz, Zakaria Saeed Mohammed Osman, Yomna Sherif Omara, Amr farouk, Ahmed Amin, Yasmine Hany Abdel Moamen Elzohery, Mohammed Gamal Ahmed Awad

**Affiliations:** 1 Department of Surgical Oncology, National Cancer Institute, Cairo University, Giza, Egypt; 2 Department of Oncoplastic Breast Surgery, Dar Al-Salam (Harmel) Cancer Hospital, Cairo, Egypt; 3 Epidemiology and Preventive Medicine Department, National Liver Institute, Menoufia, Egypt; 4 Baheya Center for Early Detection and Treatment of Breast Cancer, Giza, Egypt; 5 Radiodiagnosis Department, National Cancer Institute, Cairo University, Giza, Egypt; 6 Department of General Surgery, Faculty of Medicine, Ain Shams University, Cairo, Egypt; 7 Department of Radiotherapy, National Cancer Institute, Cairo University, Cairo, Egypt

**Keywords:** Breast reconstruction, Direct-to-implant, Ultrapro® mesh

## Abstract

**Background:**

Immediate breast reconstruction (IBR) with direct to implant (DTI) is the preferred method of reconstruction by many surgeons and patients, however, acellular dermal matrix (ADM) and other synthetic meshes are expensive especially in low- and middle-income countries.

**Aim of the work:**

To evaluate the technique, indications, aesthetic outcomes, and short and long-term complications of DTI breast reconstruction performed with Ultrapro®, a low-cost alternative mesh to ADM and other synthetic meshes.

**Methods:**

Our study is a prospective cohort study that was conducted on 133 patients who experienced IBR following nipple-sparing mastectomy (NSM) or skin sparing mastectomy (SSM) using silicone implants and Ultrapro® mesh between December 2020 and December 2023. Techniques used were either sub-pectoral or pre-pectoral, evaluating aesthetic outcome, complication rate and patient satisfaction using breast Q questionnaire.

**Results:**

We included 133 patients (141 breasts) with a median age of 39 years. Mean duration of follow up: 20.364 ± 5.39 months. The sub-pectoral and the pre pectoral techniques were used for 80 breasts and 61 breasts respectively. We used the Ultrapro® mesh in all our patients. Smooth round silicone implants were used. The overall Major complications rate was 16.3%. 8 implants (5.7%) were lost within 6 months post-operatively while 2 implants were removed in the late post-operative period (after 6 months) one due to rupture and the other due to local recurrence.

Capsular contracture Baker 3 and 4 was observed in 36 breasts (25%), 31 of them had post mastectomy radiotherapy treatment. 11 (7.8%) were managed by capsulotomies and re-insertion of the same implant.

Radiotherapy was a significant risk factors for major complications and capsular contracture with *p* value of (0.01) and (0.0001) respectively.

**Conclusion:**

DTI in properly selected patients offers excellent outcomes and patient satisfaction. The complication rate is low and improves with the experience of the surgeon. The Ultrapro® mesh is a safe, low-cost alternative to ADM or other synthetic meshes especially in low socioeconomic countries. Radiotherapy is a significant risk factor for major complications and capsular contractures.

## Introduction

Implant based reconstruction is the most common technique used post-mastectomy comprising 75% of all procedures [1]. Nipple or skin sparring mastectomy techniques achieve a natural appearance post-mastectomy and are oncologically safe and feasible [2].

Traditionally, the two-stage tissue expander followed by implant procedure is widely used for breast reconstruction. However, it impacts patients financially and emotionally [3].

The use of biological and synthetic mesh materials has significantly improved breast reconstruction techniques. While biological meshes offer advantages, their high cost has led to the development of less expensive alternatives such as Vicryl, TiLOOP®, TiLOOP® Bra, TIGR® Matrix, and Ultrapro® mesh. These non-biological materials are placed under the pectoralis major muscle to create a pocket for the breast implant following a mastectomy [4].

Ultrapro®mesh is a safe and cheaper alternative to biological matrices in DTI breast reconstruction, taking into consideration proper patient selection [4].

The Ultrapro® is a partially absorbable mesh with equal combination of monofilament lightweighted non-absorbable polypropylene with pore size of 3–4 mm and absorbable fibers of poliglecaprone-25 that absorbs within 3–4 months [4].

Several authors have discussed employing meshes to cover the lower and outer aspects of the implant in submuscular reconstructions. Others, on the other hand, have described their application in pre-pectoral reconstructions [5]. For sagging or large breasts, another technique can be used through a wise pattern incision which is done by creating a pocket superior to the breast implant where the mesh is attached to the anterior of pectoralis major muscle and inferiorly to a de-epithelialized mastectomy lower skin flap [5].

Reported complications of implant-based breast reconstruction are surgical and device-related, varying in management from conservative treatment to reoperation [1]. According to the “Mastectomy Reconstruction Outcomes Consortium study”, total complication rates range from 26.6% to 31.3%, with reoperation rates between 15.5% and 18.8%. Although severe complications are less common, surgeons must be proficient in identifying risk factors to inform decision-making and manage complications promptly. A comprehensive understanding of complication risk factors, incidence, treatments, and outcomes is crucial for effective preoperative patient counseling [1].

## Aim of the work

To evaluate the technique, indications, aesthetic outcomes, and short and long-term complications of DTI breast reconstruction performed with Ultrapro®, a low-cost alternative mesh to ADM and other synthetic meshes.

## Patients and methods

### Study design

This is a prospective cohort study that was conducted on one hundred and thirty-three patients who underwent immediate breast reconstruction by silicone implants at Baheya Center for early detection and treatment of breast cancer in Egypt from December 2020 to December 2023 following nipple sparing or skin sparing mastectomies.

All candidates for NSM or SSM were included in this study. All patients were informed about other alternative techniques. Breasts were excluded if the reconstruction was assisted by a flap or a tissue expander or a combined approach. Informed consent was obtained from all patients.

## Ethical approval

Was obtained from Baheya Research Ethics committee (BEC) at Baheya Center for Early Detection and Treatment of Breast Cancer in Egypt. Baheya IRB protocol number: 202006030013.

### Surgical technique and perioperative evaluation

Preoperatively, informed verbal and written consent were provided to all patients. Consent includes the surgical technique, explanation of advantages and possible complications including those related to the possible future adjuvant RTH.

**Fig. 1:**
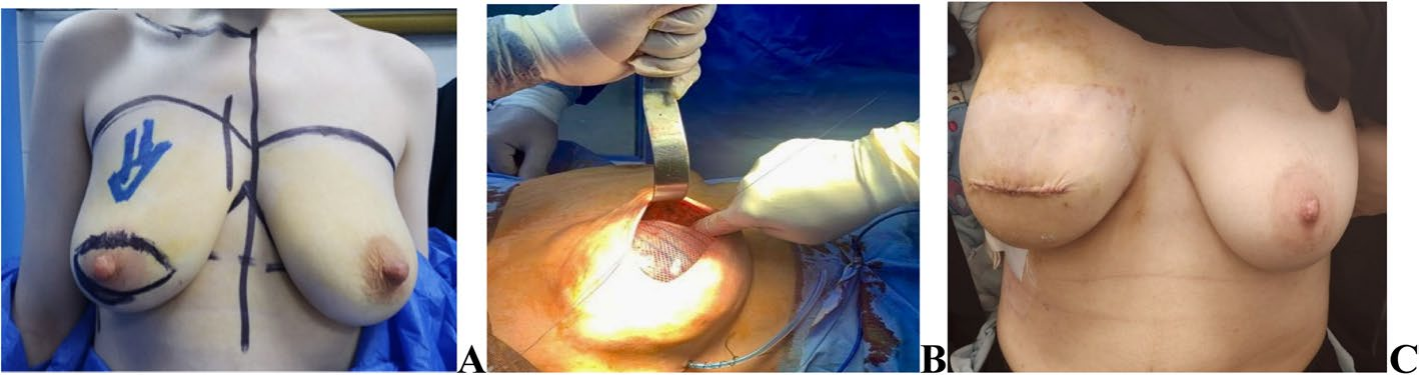
Right SSM and DTI with Elliptical peri-areolar incision (pre-pectoral approach) **A** Marking pre-operative **B** intra operative fixation of the wrapped implant at the infro-medial border of the breast **C** 2 weeks post operative

The surgical procedure was customized based on each patient's unique factors, such as their co-morbidities, preferences, cancer diagnosis, and desired outcome. Before surgery, while the patient is standing, markings are made on the breast area. The inframammary fold is marked inferiorly, and the top is determined by gently pressing the breast towards the chest to obtain the breast footprint. Medial and lateral lines were marked in the midline and the anterior axillary line respectively. (Fig. 1). Elliptical peri-areolar incision was used in skin sparing mastectomy with excision of the NAC (Fig. 1).

**Fig. 2:**
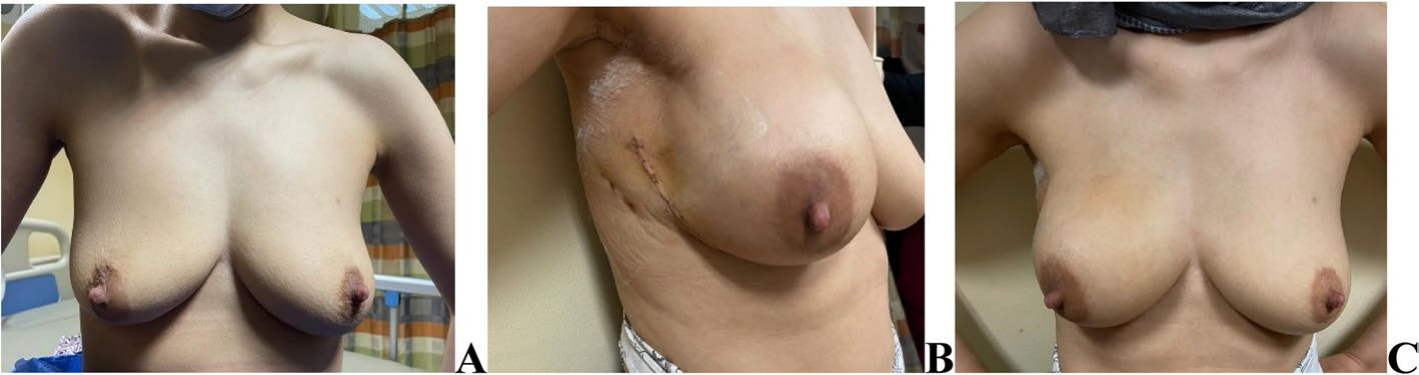
Right NSM and DTI with infro-lateral incision (pre-pectoral approach) **A** Pre-operative **B** & **C** 2 weeks post operative (lateral view & anterior view)

For nipple sparing mastectomy an inferior or infro-lateral or lateral incision were used. Large or very ptotic breasts were operated on using a wise pattern skin reduction incision. (Fig. 2).

Skin flap viability is confirmed with bleeding edges and non-dermal exposure. We proceed to either prepectoral or subpectoral technique according to breast volume. If skin flap viability is questionable, a tissue expander is placed and delayed reconstruction is to be considered.

Delayed reconstruction and patients managed by tissue expanders instead of DTI were excluded from the study.

**Fig. 3:**
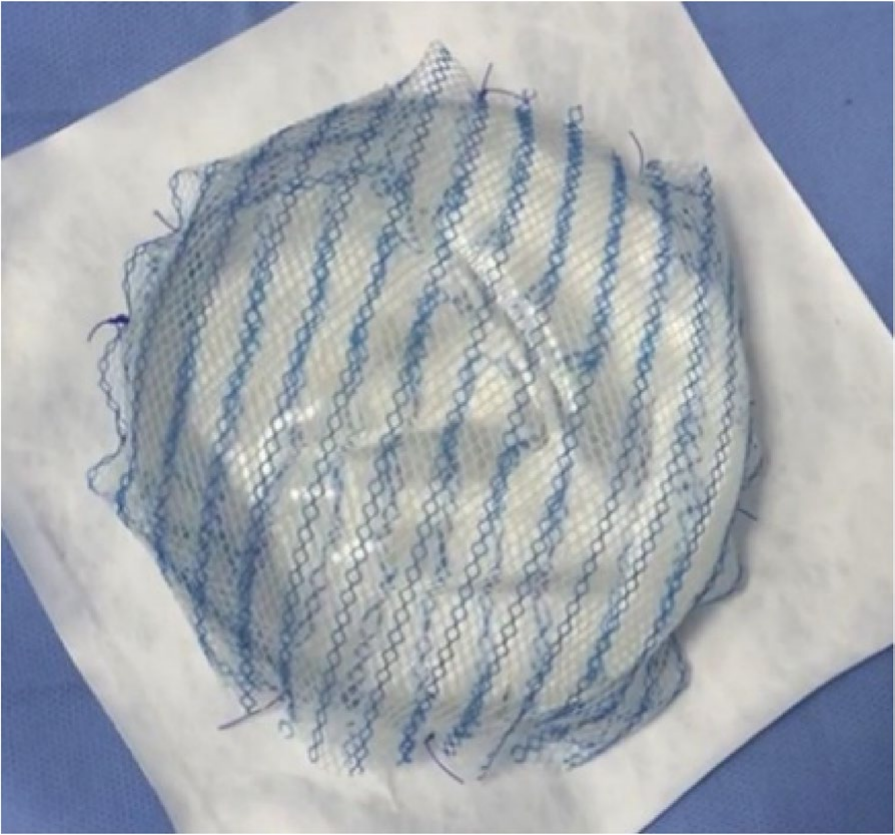
Round smooth Silicon implant completely wrapped by the ULTRAPRO® mesh

Complete wrapping of the implant with the Ultrapro® mesh pocket is performed in pre-pectoral technique where we use two meshes (two 15* 15 cm meshes or one 15* 30 cm). (Fig. 3).

**Fig. 4:**
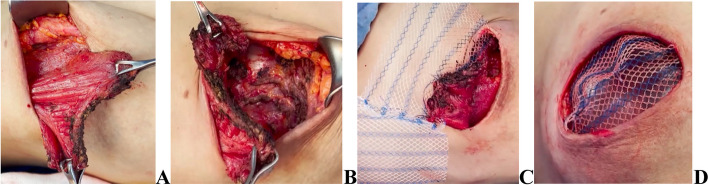
DTI sub-pectoral approach **A** cutting pectoralis muscle costal origin **B** Creation of the pocket **C** Mesh suturing to pectoralis muscle edge to create lower pole **D** Final creation of the DTI pocket

**Fig. 5:**
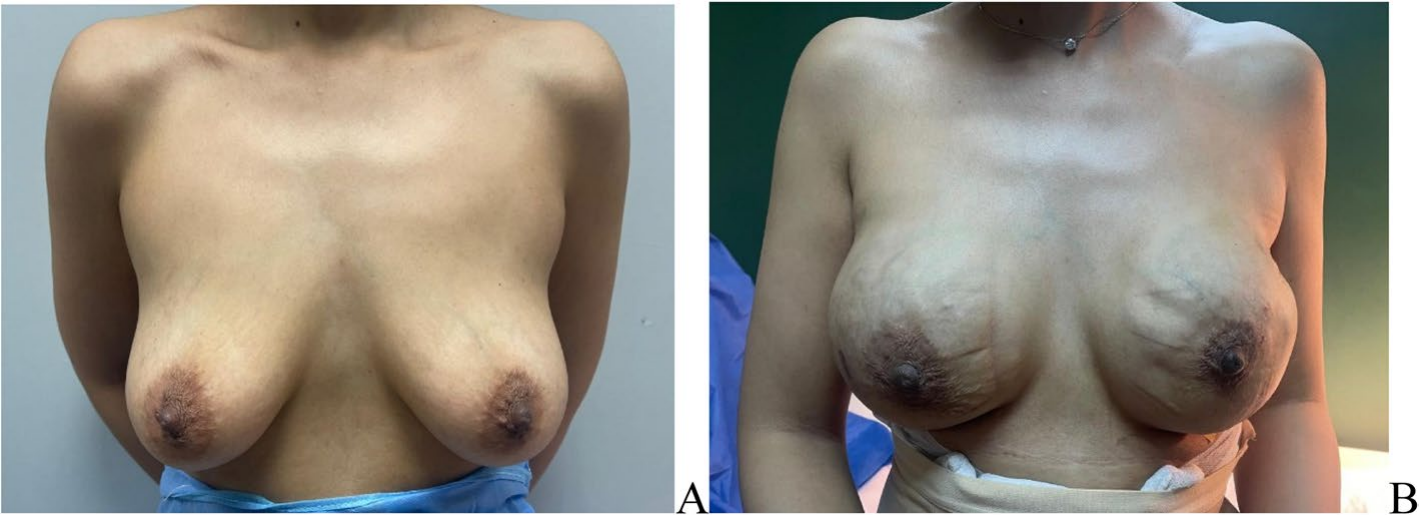
Bilateral NSM with DTI – Pre pectoral approach **A** Pre-operative **B** 2 weeks Post operative

**Fig. 6:**
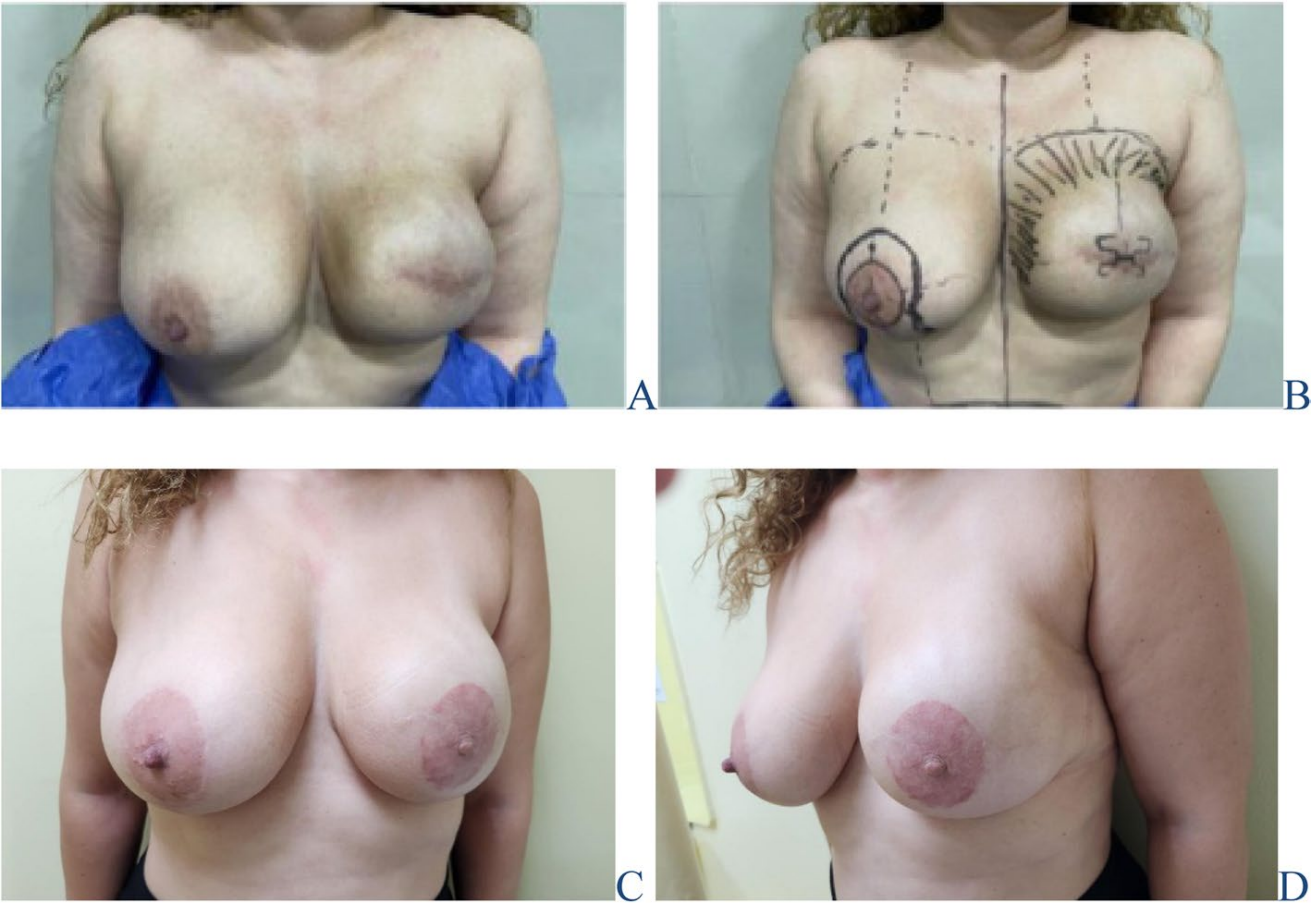
Left SSM and DTI with elliptical peri-areolar incision **A** 3 months Post operative **B** Pre-operative marking for right reduction mammoplasty, left nipple re-construction with lipofilling **C** & **D** 6 months post-operative with bilateral tattooing anterior & lateral view

In subpectoral technique a partial pocket is created by cutting the costal origin of pectoralis major muscle from lateral to medial. A 15*15 cm of Ultrapro® mesh is sewn from both edges with 2/0 vicryl sutures to the edge of the muscle and the other edge sutured to the inframammary fold. (Figs. 4, 5 and 6).

In a large ptotic breast, A de-epithelialized inferior mastectomy flap is sutured to the inferior part of the mesh then upper part of mesh is sutured to the anterior surface of pectorlais major muscle creating a prepectoral pocket for the implant.

Routinely, we place two drains after mastectomy, one in the operative bed and the other in the Axilla. 1 gm of 3rd generation cephalosporin was given to all patients at induction of anesthesia. All implants used in this study were round smooth silicone gel implants and the Ultrapro® was the only mesh employed.

### Post operative care

All patients were instructed to wear compression surgical bra until the 6th postoperative week. Drains removal was done if their output was less than 30 ml for 2 consecutive days.

All complications recorded within the study period were divided into two groups in relation to their management. The minor complications group was managed conservatively, while the major complications group was surgically managed. Both groups were further subdivided into early (within 6 months of IBR) and late (after 6 months of IBR). Such grouping was concerned with the evaluation of aesthetic outcomes and drawbacks of different reconstruction techniques post radiotherapy effect.

Skin flap necrosis, impaired or delayed wound healing or wound dehiscence were categorized as one group of complications and were documented when obviously observed. Seromas were recorded When suspected clinically or by ultrasonography. Infection was documented when clinically diagnosed or after a positive culture and sensitivity test.

Baker classification score was used to evaluate capsular contracture. Typically, patients with grade 3 and 4 capsular contracture will require intervention [6].


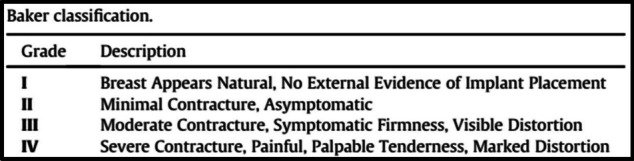


Patient's satisfaction with breast reconstruction was assessed using *Breast Q questionnaire*, which evaluates satisfaction with breast postoperatively, psychosocial well-being, satisfaction with implants, physical well-being and breast animation deformity. [7].

### Statistical analysis plan

IBM® SPSS® v28 was used for data analysis. Qualitative data is presented as frequency and relative frequency while the quantitative data is presented as mean ± standard deviation and mean (IQR). Quantitative data was checked for distribution of normality; then using Mann–Whitney U test or independent samples t-test to examine the statistical-significance between subgroups. Chi-square and Fisher’s exact tests were run to determine the difference of qualitative data among subgroups.

## Results

A total of 133 patients (141 breasts) were included in our study. Mean age of the patients was 39 years (range: 21–59 years). Mean duration of follow up 20.364 ± 5.39 months, median follow up duration 21.0 range (1.0–31.0) months. Patients with a positive family history with breast cancer were 28.9%. Invasive duct carcinoma (IDC) was the most common pathological type (61%), the 2nd most common pathology was DCIS (12.8%). Only 2 cases had benign pathology (Table 1).

**Table 1:** Basic characteristics of the study participants (*N* = 141)

	Frequency	(%)
Age (years)		
**mean ± SD**	39.31 ± 7.35	
**min–max**	21.00–59.00	
Family history		
**Negative**	94	70.6%
**Positive**	39	29.3%
Menopausal status		
**Post-menopausal**	7	5.2
**Pre-menopausal**	126	94.7
Comorbidities		
**No co-morbidities**	129	97
**DM**	2	1.5
**IHD**	2	1.5
Pathological type		
**IDC**	86	61
**DCIS**	18	12.8
**ILC**	10	7.1
**IDC + ILC**	14	9.9
**Benign phylloides**	2	1.4
**Malignant Phylloides**	1	0.7
**Others (rare pathological subtypes)**	10	7
ER (excluding phylloides breasts = 3)		
**Negative**	11	7.8
**Positive**	127	90.1
**ER not performed**	3	2.12
PR (excluding phylloides and DCIS breasts = 21 cases)		
**Negative**	6	4.2
**Positive**	114	80.9
**PR not performed**	21	14.8
HER2 (excluding phylloides and DCIS breasts = 21 cases)		
**Negative**	114	80.8
**Positive**	6	4.3
**HER2 not performed**	21	14.8
Axillary LNs (3 phylloides cases didn’t do axillary surgery)		
**Negative**	82	58.1
**Positive**	56	39.7
**NA**	3	2.12
Tumor site		
**Central**	9	6.4
**LIQ**	1	0.7
**LOQ**	1	0.7
**UIQ**	2	1.4
**UOQ**	33	23.4
**Multicentric**	88	62.4
**Multifocal**	7	5
Tumor size		
**mean ± SD**	3.84 ± 2.67	
**min–max**	1–15 cm	

Ninety-nine of breasts reconstructions (70.2%) were SSM, thirty-three (23.4%) were NSM and 9 (6.4%) were skin reducing mastectomy (SRM). All axillary surgeries whether SLNB (53.9%) or ALND (43.9%) were done using the same mastectomy incision. 3 breasts with benign and malignant phylloides had no axillary surgery performed (Table 2).

**Table 2:** Illustrating the surgical details and additional therapy of the study participants

	**Frequency**	(%)
Operation		
**NSM**	33	23.4
**SRM**	9	6.4
**SSM**	99	70.2
axillary surgery		
**ALND**	62	43.9
**SLNB**	76	53.9
**No Axillary surgery**	3	2.1
Laterality		
**LT**	73	51.8
**RT**	52	36.9
Bilateral	8 (16 breasts)	11.3
Cup size		
**A**	5	3.5
**B**	40	28.4
**C**	57	40.4
**D**	39	27.7
Mesh		
Ultrapro®	141	100
Site		
**Pre-pectoral**	61	43.3
**Sub-pectoral**	80	56.7
implant size		
**mean ± SD**	436.13 ± 91.15	
**min–max**	200.0- 690.00	
drainage removal /days		
**mean ± SD**	12.63 ± 4.31	
**min–max**	5.00- 33.00	
Chemotherapy (133 patients)		
**Not indicated**	37	26.2%
**neoadjuvant**	56	39.7%
**adjuvant**	40	28.3%
Hormonal treatment		
	119	89.4%
Radiotherapy (per breast)		
yes	77	54.6%
no	64	45.4%

Pre-pectoral and sub pectoral techniques were performed for sixty-one breasts (43.3%) eighty breasts (56.7%) respectively (Table 2).

Mean implant size was 436 with range between 200 and 690. all implants were high cohesive silicone gel of the smooth round type either moderate or high profile. No textured or saline filled implants were used in this study.

About 30% of patients received adjuvant CTH while 39.7% received neoadjuvant CTH and in 26.2% of patients CTH was not indicated. 54.6% of patients received adjuvant RTH (Table 2).

### Complications

The overall rate of complications was recorded in 43 breasts (30.4%), 20 breasts (14.19%) were within the early post-operative period while 23 (16.3%) were after 6 months of breast reconstruction.

**Fig. 7:**
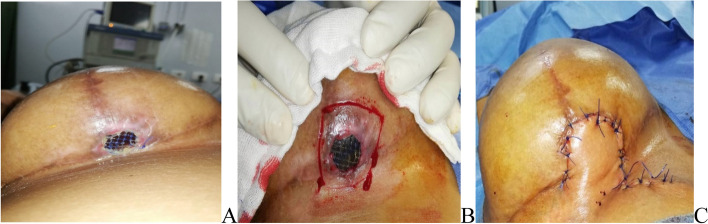
**A** Post operative Skin flap necrosis in inverted T incision, **B** Managed by trimming of edges, excision of mesh exposed part, **C** Rotational skin flap for the final repair

In the early post-operative period, seroma was recorded in 5 breasts and hematoma was observed in 1breast (4.2%). The 6 cases were treated conservatively by aspiration. Skin flap necrosis was diagnosed in 7 breasts, 6 of them were in the late post-operative period and required re-operation where one of them was treated by trimming of edges, excision exposed part of the mesh and rotational skin flap (Fig. 7). Infection and superficial sloughing were observed in 14 breasts (9.9%), 10 of which were early and treated with repeated dressings and empirical antibiotics followed by definitive antibiotics after culture and sensitivity, the other 4 cases were presented late where 2 of them required debridement and 2ry suturing and 2 needed explantation. Four breasts (2.8%) observed with superficial sloughing, 3 of which treated conservatively while 1 needed debridement and 2ry suturing.

**Fig. 8:**
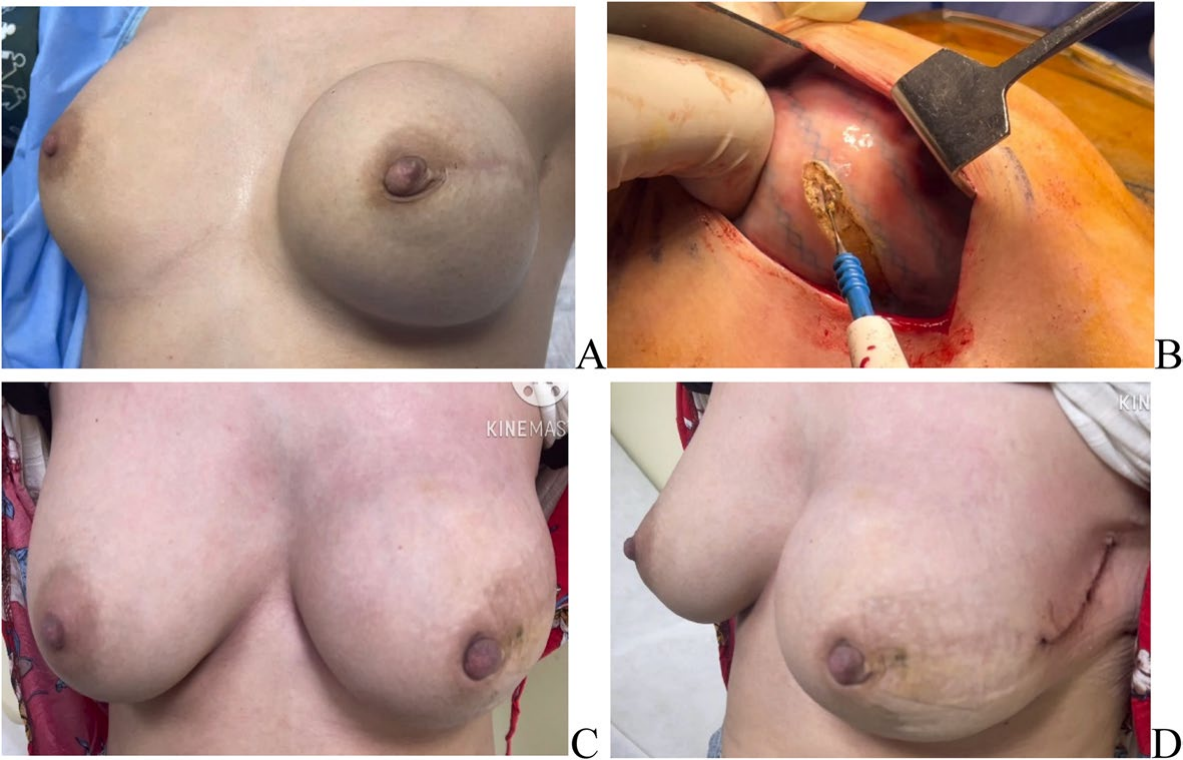
Post left NSM and DTI with capsular contracture grade 4 [**A** Grade 4 capsular contracture. **B** intra-operative capsulotomy. **C** & **D** Post capsulotomy (anterior & lateral view)]

Necessity for surgical intervention due to capsular contracture Baker 4 was done in 11 breasts (7.8%) where capsulotomies or capsulectomies with re-insertion of the same implant were performed (Fig. 8). All of were in the late post operative received post mastectomy radiotherapy.

One case presented with implant rupture and needed re-operation and exchange of implant.

A total of 9 implants (6.4%) were lost due to complications, 8 of them in the early post-operative period due to infection, superficial sloughing or skin flap necrosis and one implant was removed in the late complications group due to implant rupture.

Major complications were recorded in 23 breasts (16.3%) which represents cases requiring re-operation for debridement, 2ry suture, implant removal or capsulotomies.

During our study period, we lost 10 implants (7%), 9 of them due to complications and 1 was due to recurrence in the operative bed (Table 3).

**Table 3:** Complications encountered by the study participants

	**Minor complications**			**Major complications (requiring surgery)**		
	***N*** **= 20 (14.19%)**			***N*** **= 23(16.3%)**		
	**Early Minor complications:**	20	14.19%	**Early major complications:**	**11**	**7.8%**
**Early**	Infection and superficial sloughing	10	7.10%	Infection and superficial sloughing	4	2.8%
	Skin flap necrosis ± infection	1	0.7%	Skin flap necrosis ± infection	6	4.2%
	Superficial sloughing only	3	2.1%	Superficial sloughing only	1	0.7%
	Seroma or hematoma	6	4.2%	-	-	-
	Management			Management		
	AB + repeated dressings	14	9.9%	Re-surgery for Debridement & 2ry suturing	3	2.10%
	Aspiration	6	4.2%	Implant removal	8	5.70%
	**Late minor complications:**	-	-	**Late major complications:**	**12**	**8.5%**
**Late**	-	-	-	Capsular contracture Baker 4 requiring surgery	11	7.8%
	-	-	-	Implant rupture	1	0.7%
	Management			Management		
	-	-	-	Capsulotomy/ capsulectomy	11	7.8%
	**-**	**-**	**-**	implant removal for implant rupture case	1	0.7%

### Correlation between risk factors and complications (Table 4)

**Table 4:** Risk factors for complications

**Studied variable**		**No major complications (*n*=118)**		**Major complications (*n*=23)**		***P*** ** value**
		No.	%	**No**	**%**	
**Axillary surgery**	**ALND (n=62)**	47	75.8%	15	24.2%	**0.17**
	**SLNB (n=76)**	66	86.8%	10	13.1%	
	**No Axillary surgery (n=3)**	3	100%	0	0.0%	
**Implant size**	**mean ± SD**	423.53± 93.76		446.90± 88.04		**0.27**
**SITE**	**Pre-pectoral (61)**	50	82.00%	11	18.00%	**0.06**
	**Sub-pectoral (80)**	68	83.80%	12	16.30%	
**Drainage removal /days**	**mean ± SD**	12.03±3.61		13.39±4.97		**0.06**
**Radiotherapy**	N**o (64)**	59	92.18%	5	7.8%	**0.01**
	Y**es (77)**	59	76.60%	18	23.3%	
**Chemotherapy**	**Chemotherapy not indicated (n= 37)**	34	91.8%	3	8.1%	**0.16**
	**Neoadjuvant Chemotherapy (n=56)**	43	74.10%	13	23.2%	
	**Adjuvant chemotherapy (n=40)**	33	82.5%	7	17.5%	

#### Drain removal

There is a statistically significant difference between complications rate and the drainage removal time. The median (IQR) of patients suffered from complications was 13.39 (+ -4.97) days which is higher than of patients did not suffer from complications (12 + -3.61 days) (Table 4.).

#### Size and site of implant and mesh usage

The implant size, site of implant is not statistically significant factors for complications (Table 4.).

#### Adjuvant treatment

There is no statistically significant relation between rate of complications and chemotherapy either adjuvant or neo-adjuvant. However, patients receive radiotherapy are at higher risk for complications (*P* value 0.01) (Table 4.).

Upper pole visibility was observed among 20 breasts and mainly in the pre-pectoral group (22.9%) (Table 5).

**Table 5:** Relation between site of implant and its drawbacks

Studied variable	Pre pectoral		Sub pectoral		*p*-value
	**(** ***n*** **= 61)**		**(** ***n* = 80)**		
	**No**	**%**	**No**	%	
**Upper pole visibility**					
No	47	77.10%	74	92.50%	**0.009**
Yes	14	22.90%	6	7.50%	
**Implant removal**					
No	53	86.90%	78	97.50%	**0.04**
Yes	8	13.10%	2	2.50%	

Out of the 141 breasts included in this study, 10 breasts underwent implant removal 8 implants in the prepectoral group and 2 in the subpectoral group (one due to implant rupture and one due to local recurrence).

No statistical significance was found between pre pectoral and subpectoral techniques in terms of complications. (Table 6).

**Table 6:** Relation between site of implant and complications

Studied variable	Pre pectoral		Sub pectoral		*p*-value
	**(** ***n*** **= 61)**		**(** ***n*** **= 80)**		
	**No**	**%**	**No**	**%**	
**Early Minor 20**	9	45%	11	55%	**0.81**
**Early Major 11**	6	54.5%	5	45.4%	
**Late Major 12 (11 breasts with capsular contracture Baker 4)**	5	41.6%	7	58.3%	

Capsular contracture Baker 3 and 4 was observed in a total of 36 breasts (25%) where 11 of them were Baker 4 and managed surgically while 25 breasts didn’t require management and were followed up. 31 of the 36 capsular contractures (86%) had post mastectomy radiotherapy treatment (Table 7).

**Table 7:** RTH as a risk factor for complication in direct to implant

Studied variable	Pre pectoral/ NO radiotherapy		Pre pectoral/ with radiotherapy		Sub pectoral/ No radiotherapy		Sub pectoral/ with radiotherapy		*p*-value
	**(** ***n*** **= 26)**		**(** ***n*** **= 35)**		**(** ***n*** **= 38)**		**(** ***n*** **= 42)**		
	**No**	**%**	**No**	**%**	**No**	**%**	**No**	**%**	
**Capsular contracture**	3	11.5%	16	45%	2	5%	15	35%	0.0001

We took some measures to decrease the rate of capsular contracture including employing complete aseptic techniques intraoperatively, preventing seroma formation by delaying drain removal until the output was less than 30 ml, using prophylactic antibiotics to prevent biofilm formation, and using smooth silicone implants instead of textured ones.

Taking into consideration that 31 of the 36 cases with capsular contracture (86%) had post-mastectomy radiotherapy.

### Aesthetic outcome

Breast Q questionnaire was used to assess the aesthetic outcome of different DTI techniques using presented in (Table 8).

**Table 8:** Summary of the score of different domains of Breast Q

Studied variable	Psychosocial well benign	Satisfaction with breasts (post operative)	Satisfaction with Implants	Physical wellbeing (chest)	Breast animation deformity
Mean ± SD	66.51 ± 26.63	66.02 ± 6.11	6.44 ± 1.44	69.32 ± 21.0	71.70 ± 15.59
Median (IQR)	74.0 (53–83)	67.0 (58–75)	6.00 (6.0–8.0)	72.0 (53–81)	70.0 (62–79)
Min–max	32—100.00	12- 100.00	2.00- 8.00	20- 100.00	11.0- 100.00

#### Breast Q

Patients who experienced complications after breast surgery reported significantly lower scores of psychological well-being and satisfaction with their new breasts compared to those who did not experience complications. The 117 patients without complications reported significantly higher satisfaction scores (Table 9).

**Table 9:** Relation between breast Q and complications

	**No major complications**	**Major complications**	***p*** **-value**
	**(** ***n*** **= 117)**	**(** ***n*** **= 23)**	
	**Median (IQR)**	**Median (IQR)**	
**Psychosocial well benign**	74.0 (61.0–85.0)	47.5 (47.0 -68.2)	** < 0.001**
**Satisfaction with breasts (post operative)**	67.0 (58.0- 76.5)	59.5 (50.25–75.7)	**0.26**
**Satisfaction with Implants**	6.5 (6.0–8.0)	6.0 (6.0- 7.25)	**0.27**
**Physical wellbeing (chest)**	72.0 (55.0–85.0)	68.0 (43.75–74.0)	**0.22**
**Breast animation deformity**	73.0(62.0–79.0)	69.0 (58.75–76.0)	**0.21**

No statistical difference was observed between Breast Q scores between pre-pectoral and subpectoral cases (Table 10).

**Table 10:** Relation between site of implant and breast Q

Studied variable	Pre pectoral	Sub pectoral	*p*-value
	**(** ***n*** **= 61)**	**(** ***n*** **= 80)**	
	**Median (IQR)**	**Median (IQR)**	
**Psychosocial well benign**	74.0 (55–87)	74.0 (48–83)	**0.46**
**Satisfaction with breasts (post operative)**	71.0 (58- 78)	65.0 (54- 75)	**0.33**
**Satisfaction with Implants**	7.0 (6–8)	6.0 (6–8)	**0.27**
**Physical wellbeing (chest)**	72.0 (56–85)	68.0 (50–80)	**0.37**
**Breast animation deformity**	73.0 (62- 84)	70.0 (62- 76)	**0.79**

## Discussion

Multiple options are available for breast reconstruction following mastectomy, implant-based techniques are currently the most common [8, 9]. This study aimed to evaluate the technique, indications, aesthetic outcomes, and short and long-term complications of DTI breast reconstruction performed with Ultrapro®, a low-cost alternative mesh to other biological matrices as the ADM and other synthetic meshes.

The overall complications rate was 30.4%, 14.19% were minor complications that only required conservative management. Surgical intervention was required in 16.3%. the most common complication was infection with superficial sloughing observed in 14 breasts (9.9%) while skin flap necrosis ± infection in 7 cases (4.9%), superficial sloughing in 4 breasts (2.8%), seroma in 5 breasts (3.5%), hematoma in 1 (0.7%) Capsular contracture requiring surgery in 11 breasts (7.8%) and implant rupture in 1 case (0.7%).

The risk of complications is significantly higher in cases treated with adjuvant radiotherapy. Out of 77 breasts treated with radiotherapy, 18 breasts (23.3%) presented with major complications requiring re-operation compared with 5 (7.8%) out of 59 breasts not irradiated (*p* = 0.01) and this was validated by Spear et al., who used ADM in IBR [10].

In 2017, Sigalove et al. reported less than 5% of aesthetic complications after pre-pectoral reconstruction with ADM as capsular contracture, implant malposition, and rippling. Their complication rate was 9.1%: 4.5% infections, 2.5% necrosis, and 2.0% seromas [11].

We had implant explanation in a total of 10 cases, 9 (6.3%) of which were due to major complications as infection, superficial sloughing or skin flap necrosis and one due to implant rupture in which the implant was exchanged. The 10th lost implant was due to local recurrence in the operative bed.

A 2019 study by Potter and colleagues involved over 2,000 women in the UK to assess the immediate safety of breast reconstruction using implants with or without mesh. The implants were placed either pre pectoral of or sub pectoral. After three months, nearly 10% of patients lost their implants, 18% were readmitted to the hospital, 18% required re-operation, and 25% experienced infections. Importantly, the use of mesh, whether biological or synthetic, did not affect these complication rates [12].

Pukancsik et al., in a study in the National Institute of Oncology in Budapest included 102 patients (174 breasts) reconstructed using implant and Ultrapro® mesh, [4]. Pukancsik et al. reported complications in 32 cases (18.3%), 12 (6.9%) of them were minor complications while 20 cases (11.4%) suffered major complications requiring surgical intervention. Out of 8 infections (4.5%), 3 of them (1.7%) required re-operation without removal of the mesh or the implants. 2 cases had hematoma collection (1.2%). Out of 9 seroma Cases (5.1%), 5 required re-operation due to chronicity [4].

Finally, they reported implant extrusion in 7 cases (4%) due to skin necrosis. Implant malposition reported in 4 cases (2.3%) and Capsular contracture G3 and 4 in 2 cases (1.2%) treated with capsulectomies, implant removal and delayed breast reconstruction using Latissimus Dorsi Flap [4].

They concluded that synthetic, partially absorbable Ultrapro® mesh showed encouraging results in DTI IBR over a long-term period of evaluation and offers a potentially safe, effective, and less expensive alternative to biological matrices [4].

Choosing the right breast implant is crucial for achieving optimal aesthetic outcomes. Using an implant with insufficient width can create an undesirable indentation on the side of the chest. Having a range of implant sizes available during surgery is helpful to ensure the best fit [4].

J. Kalstrup et al. in 2021 operated on 232 breasts with ADM assisted DTI IBR. They reported 34% of patients developing one or more complications where necrosis was seen in 39 breasts (17%) where 16 of them required surgery. 14 patients with infection (9%), 12 (8%) with seroma formation and 6 (4%) with hematomas within the first 6 months post operative. While they observed late seroma formation in 8 patients. Twenty patients (13%) needed explanation due to hematoma, infection or necrosis where 9 of them had implant loss (6%) [13].

Kalstrup et al. didn’t find statistical significance between post operative radiotherapy and complications. However, they reported a strong association between explanation and *pre-operative* radiotherapy (*P* = 0.045) which doesn’t correlate to our study [13].

In 2019, Lohmander et al. in a Dutch multicenter RCT randomized 142 women to DTI breast reconstruction with ADM or two-staged implant-based reconstruction without ADM. 11% of complications were found in the ADM group in comparison to 4% in the non-ADM group. 8% Wound infection vs. 2% of the in ADM and non-ADM respectively. Skin necrosis was reported in 12% vs. 1%, and wound dehiscence in 9% vs. 0%, respectively [14].

As regards to capsular contracture, our study found a strong statistical relation between post mastectomy radiotherapy and capsular contracture either pre pectoral or sub-pectoral approach (*P* = 0.0001). capsular contracture was diagnosed in 36 breasts, 31 of them (86%) were treated with post mastectomy radiotherapy.

Hammond et al. in 2020 studied the incidence and risk factors of capsular contracture post mastectomy and implant-based re-construction and found a strong association with post mastectomy RTH and capsular contracture development (*P* = 0.001). They reported the rate of capsular contracture among patients receiving RTH was 18.7% which correlates with our results. While on the other hand, the non-irradiated patients with capsular contracture were 7.5%. They also mentioned a strong association between capsular contracture and postoperative hematoma (*p* = 0.047) and neoadjuvant chemotherapy (*P* = 0.004) [6].

Despite facing many challenges during our experience with DTI such as: using a low cost-effective Ultrapro® mesh, implant sizes and dealing with large sized breasts and high BMI in most of our Patients, we managed to obtain satisfactory breast-Q scores namely in the following domains: Psychosocial well benign: median 74 (mean + -SD 66.51 ± 26.63), Satisfaction with breasts post operative: 67 (66.02 ± 6.11), Satisfaction with Implants: 6 (6.44 ± 1.44), Physical wellbeing (chest) 72 (69.32 ± 21.0), Breast animation deformity 70 (71.70 ± 15.59).

There is statistically significant difference between complicated and uncomplicated cases as regards the Psychosocial well benign Satisfaction with breasts (post operative).

### Study Limitations

We enrolled all patients who came to our hospital and matched with the study selection criteria. But we recommend to do larger multi-centric study in future in collaboration with other institutions and hospitals outside Egypt.

## Conclusion

DTI in properly selected patients offers excellent outcomes and patient satisfaction. The complication rate is low and improves with the experience of the surgeon. The Ultrapro® mesh is a safe, low-cost alternative to ADM or other synthetic meshes especially in low socioeconomic countries. Radiotherapy is a significant risk factor for major complications and capsular contractures.

## Data Availability

No datasets were generated or analysed during the current study.

## Abbreviations

AB: Antibiotics
ADM: Acellular dermal matrix
ALND: Axillary lymph node dissection
BER: Baheya research ethical committee
IBR: Immediate Breast reconstruction
CTH: Chemotherapy
DCIS: Ductal carcinoma in situ
DM: Diabetes mellitus
DTI: Direct-To-Implant
IDC: Invasive duct carcinoma
IHD: Ishemic heart disease
ILC: Invasive lobular carcinoma
IOQ: Inner outer quadrant
LIQ: Lowe inner quadrant
NAC: Nippe areola complex
NSM: Nipple sparring mastectomy
RTH: Radiotherapy
SLNB: Sentinel lymph node biopsy
SRM: Skin reducing mastectomy
SSM: Skin sparring mastectomy
UIQ: Upper inner quadrant
UOQ: Upper outer quadrant
